# IL-6 blockade in systemic juvenile idiopathic arthritis – achievement of inactive disease and remission (data from the German AID-registry)

**DOI:** 10.1186/s12969-018-0236-y

**Published:** 2018-04-05

**Authors:** M. Bielak, E. Husmann, N. Weyandt, J.-P. Haas, B. Hügle, G. Horneff, U. Neudorf, T. Lutz, E. Lilienthal, T. Kallinich, K. Tenbrock, R. Berendes, T. Niehues, H. Wittkowski, E. Weißbarth-Riedel, G. Heubner, P. Oommen, J. Klotsche, Dirk Foell, E. Lainka

**Affiliations:** 1 Department of Pediatric Rheumatology, University Children’s Hospital Essen, Hufelandstr. 55, 45147 Essen, Germany; 2 German Center for Pediatric and Adolescent Rheumatology, Garmisch-Partenkirchen, Germany; 3 Department of Pediatrics, Asklepios Clinic Sankt Augustin, Centre for Pediatric Rheumatology, Sankt Augustin, Germany; 4 Center for Pediatric and Adolescent Medicine/Pediatric Rheumatology, University Hospital Heidelberg, Heidelberg, Germany; 5 Department of Pediatrics, Ruhr-University Bochum, Bochum, Germany; 6 Department of Pediatric Pneumology and Immunology and Center for Chronically Sick Children of the Charité, Charité University Medicine Berlin, Berlin, Germany; 7 Department of Pediatric Pneumology, Allergology and Immunology, RWTH Aachen University, Aachen, Germany; 8 Department of Pediatric Rheumatology, St. Marien’s Children’s Hospital Landshut, Landshut, Germany; 9 HELIOS Children’s Hospital Krefeld, Pediatric Immunology and Rheumatology, Krefeld, Germany; 10 Department of Pediatric Rheumatology and Immunology, University of Münster, Münster, Germany; 11 Department of Pediatric Rheumatology, University Children’s Hospital Hamburg-Eppendorf, Hamburg, Germany; 12 Children’s Hospital Dresden-Neustadt, Dresden, Germany; 13 Department of Pediatric Oncology, Hematology and Clinical Immunology, Center for Child and Adolescent Health, Medical Faculty, Heinrich-Heine-University Düsseldorf, Düsseldorf, Germany; 14 German Rheumatism Research Centre Berlin, Berlin, Germany; 15 Department of Pediatric and Adolescents medicine, Medical faculty, University Hospital of Cologne, Cologne, Germany

**Keywords:** Systemic juvenile idiopathic arthritis, Autoinflammatory disease, Proinflammatory cytokines, Interleukin-6, Tocilizumab

## Abstract

**Background:**

Systemic juvenile idiopathic arthritis (sJIA) is a complex disease with an autoinflammatory component of unknown etiology related to the innate immune system. A major role in the pathogenesis has been ascribed to proinflammatory cytokines like interleukin-6 (IL-6), and effective drugs inhibiting their signaling are being developed. This study evaluates sJIA patients treated with the IL-6 inhibitor tocilizumab (TCZ) concerning clinical response rate, disease course and adverse effects in a real-life clinical setting.

**Methods:**

In 2009 a clinical and research consortium was established, including an online registry for autoinflammatory diseases (AID) (https://aid-register.de). Data for this retrospective TCZ study were documented by 13 centers.

**Results:**

From 7/2009 to 4/2014, 200 patients with sJIA were recorded in the AID-registry. Out of these, 46 (19 m, 27 f, age 1–18 years) received therapy with TCZ. Long term treatment (median 23 months) has been documented in 24/46 patients who were evaluated according to Wallace criteria (active disease 6/24, inactive disease 5/24, remission 13/24 cases). Under observation co-medication were used in 40/46 cases. Adverse events were reported in 11/46 patients. The clinical response rate (no clinical manifestation, no increased inflammation parameters) within the first 12 weeks of treatment was calculated to be 35%.

**Conclusion:**

Out of 200 sJIA children reported in the German AID-registry, 46 were treated with TCZ, showing a clinical response rate of 35% during the first 12 weeks, and inactive disease and/or remission under medication in 75% after one year. Adverse events were seen in 24% and severe adverse events in 4%.

**Trial registration:**

The AID-Registry is funded by the BMBF (01GM08104, 01GM1112D, 01GM1512D).

## Background

Systemic juvenile idiopathic arthritis (sJIA) is a complex disease with an autoinflammatory component of unknown origin. sJIA is characterized by daily spiking fever which persists for at least 2 weeks, arthritis, rash, serositis, lymphadenopathy or hepato(spleno)megaly [1]. Currently, sJIA is classified by the International League of Associations for Rheumatology (ILAR) as a subtype of juvenile idiopathic arthritis (JIA) and represents about 10–20% of all JIA in the Caucasian population [2]. The ILAR classification criteria for sJIA have been criticized in the last years. Various initiatives developed new criteria for pediatric population (CARRA, PRINTO, GKJR) [3–5]. Today the most severe complication, macrophage activation syndrome (MAS), is one of the major causes of mortality in pediatric rheumatology [6]*.* sJIA is associated with erosive arthritis, growth retardation, cardiovascular and pulmonary morbidity as well as amyloidosis. The prevalence of amyloidosis was approximately 1–2% until the 1990s and decreased continuously in the last years [7].

sJIA is based on abnormalities in the innate immune system leading to an activation of immunocompetent cells, e.g. phagocytes, with the release of proinflammatory interleukins (ILs) like IL-1, IL-6, IL-18 and proinflammatory S100-proteins [1]. Biologicals like IL-1 inhibitors and tocilizumab (TCZ) are both approved for the treatment of sJIA [8–12]. TCZ is a humanized monoclonal antibody directed against the IL-6 receptor. It is the first biological drug being approved for the treatment of sJIA in the European Union 2011 for use alone or in combination with methotrexate (MTX), in children older than 2 years. Its effectiveness treating children younger than 2 years is under investigation [13]. By 2013, the proportion of patients with sJIA who were treated with biologicals has increased to 20% in Germany [14]. Aims: We present the first follow-up results of the AID-registry work concerning clinical response rates (no clinical manifestation, normal inflammation parameters), achievement of inactive disease and remission (Wallace criteria), disease courses, inflammatory parameters, outcome, concomitant medication and adverse effects from 46 out of 200 sJIA patients treated with TCZ in Germany in a real-life clinical setting.

## Methods

### Translational AID-net

The AID-registry is part of the AID-Net (Network for autoinflammatory diseases), a research initiative funded by the German Federal Ministry of Education and Research. Patient data are documented following a pseudonymisation procedure in an online registry (https://aid-register.de); additionally, patient material is collected and stored in a biomaterial bank for serum and plasma [15]. 

### Patients

From 2009 to 2014, more than 200 patients with new onset or already established diagnosis of sJIA, which was confirmed by pediatric rheumatologist, were included in the AID-registry. Demographic information, clinical data, and blood samples (serum and EDTA blood) for biomarkers and genetic analysis are collected at study enrollment and follow-up visits. Generally one visit per quarter was scheduled for each patient accounting for an average of 4 visits per year in all sJIA patients. A total of 46/200 (23%) sJIA children were treated with TCZ and longitudinally documented in follow-up. Patients treated with TCZ provided follow-up visits at week 5 and week 12 after treatment start. These patients were available for analysis of the clinical response at week 12 after start of TCZ treatment. The clinical response rate under TCZ at week 12 measured the effectiveness of TCZ and was defined as follows: no symptoms and normal inflammation parameters at that time. Remission after 12 months was evaluated based on Wallace criteria, and therefore we differentiate between active disease (AD), inactive disease (ID) and clinical remission on medication (CRM). Wallace et al. defined ID by meeting the following criteria: no active arthritis, no fever, no exanthema, no serositis, no splenomegaly, no lymphadenopathy, no active uveitis, normal ESR and CRP, no disease activity in physician’s report. CRM was defined as ID for at least 6 months. Active disease (AD) described a visit with symptoms (see above) and increased inflammatory parameters [16]. CRP, ESR (after 1 and 2 h) and leukocytes were measured and the number of patients with normal levels was determined. Different clinical phenotypes were defined: monocyclic (MC) means a persistent flare, after one episode the disease is inactive; polycyclic (PC) are recurrent flares (active disease changed with inactive disease); polyarticular (PA) are flares with arthritis > 4 joints. The concomitant medication before and during TCZ application was analyzed in detail.

### Inclusion criteria (Fig. 1)

We included sJIA patients who fulfilled ILAR classification criteria [17], but also other definitions for sJIA confirming diagnosis by treating pediatric rheumatologists [3, 18–20]. For study inclusion, a minimum number of 2 visits per year were necessary for each patient. Patients fulfilling these inclusion criteria were included from the following AID-Net centers: Garmisch-Partenkirchen (*n* = 23), Essen (*n* = 3), Heidelberg (*n* = 3), St. Augustin (n = 3), Aachen, Berlin, Bochum, Dresden, Duesseldorf, Hamburg, Krefeld, Landshut and Muenster (each *n* = 1–2).

**Fig. 1:**
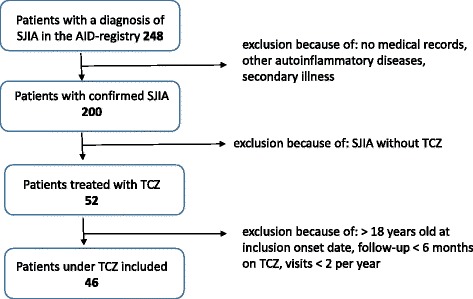
Flow chart for the inclusion criteria

### Statistical analysis

Descriptive analyses included mean and range for continuously distributed parameters as well as absolute and relative frequencies for categorical data. The number of adverse events was reported by 100 years under TCZ treatment. The confidence interval for the rate per 100 treatment years was estimated by exact Poisson intervals. The likelihood to achieve a clinical response under TCZ at week 12 was analyzed by logistic regression analyses. Additionally, clinical responses at various visits (response at < 5 weeks, 6–12 weeks, 13–24 weeks, no response at > 24 weeks) were correlated with disease activity at the last available visit in 2014. Logistic regression analyses were conducted in STATA 12.1 (StataCorp. 2011. Stata Statistical Software: Release 12. College Station, TX: StataCorp LP).

### Ethics commission

The AID-registry has been approved by the ethics committees and the data protection responsibles at the University of Duisburg-Essen and Muenster, as well as the Medical Association Nordrhein in Duesseldorf. Parents, children between 9 and 13 years of age and young patients aged ≥14 years provided informed consent.

## Results

### Patient cohort

Forty-six children (19 m, 27 f) with a median age of 11 (range 2–21) years at their last documented visit (up to 04/2014) were included in the analyses. Age of first symptoms was 4 (range 0.6–16) years. Altogether, 680 visits were analyzed. Therapy with TCZ was started at the age of 9 (range 1–18) years. Duration from diagnosis to TCZ start was 24.5 (1–188) months. 28/46 (61%) had a Caucasian origin (Germany, Austria, Poland, Italy, Croatia), 4/46 (9%) were from Turkey and 6/46 (13%) from other East European (Bulgaria, Albania) and Asian countries and 8/46 (17%) of unknown or mixed ethnicity. 34/46 (74%) patients fulfilled ILAR classification criteria, 12/46 (26%) patients did not fulfill ILAR criteria but other definitions for sJIA and diagnosis was confirmed by pediatric rheumatologists [3, 18–20].

### Clinical and laboratory parameters

Symptoms at diagnosis of sJIA and at the start of TCZ treatment did not differ significantly. Fever, lymphadenopathy, abdominal pain, hepatomegaly, nausea and vomiting were seen less often at TCZ start, whereas morning stiffness, myalgia, diarrhea, arthralgia and arthritis were observed more frequently. Before TCZ CRP was increased (90 (range 0.1–320) mg/l). Laboratory parameters (CRP, ESR, leukocytes) before diagnosis and under IL-6 inhibition revealed that monocyclic (MC) courses of sJIA, before initiating therapy with TCZ, showed higher CRP and ESR levels than polycyclic (PC) and polyarticular (PA) courses. After TCZ initiation CRP and ESR levels declined irrespective of the disease course. In fact, 27/46 (59%) patients receiving TCZ showed no measurable CRP during therapy or during a clinically diagnosed flare. 10/46 (22%) showed no measurable CRP within the first 4 weeks after starting TCZ.

### Response rate

The median time under TCZ exposure was 14.9 (range 1–48) months for the 46 sJIA patients. In total, 18 (39.1%) patients achieved an inactive disease and 14 (30.4%) a state of remission on medication according to the Wallace criteria. Therefore, a favourable outcome under TCZ treatment was reported for 32/46 (69.6%) of the patients in the registry. Remission on medication was additionally analyzed in a subgroup of patients who were treated with TCZ for at least 12 months. 24/46 sJIA patients (52%) who received TCZ for a median of 23 (range 12–48) months were available for this analysis. The state of remission on medication was achieved for 13/24 (54.2%) sJIA patients, and additional 5/24 (20.8%) were in a state of inactive disease, amounting 18/24 (75%) patients with favourable outcome.

Additionally, clinical response rates within the first 12 weeks of treatment have been estimated in all 46 patients. 16/46 (35%) patients showed an effectiveness of TCZ with no clinical manifestation and normalized inflammation parameters after 12 weeks of treatment. Comparison of initial response (at < 5 weeks, 6–12 weeks, 13–24 weeks, no response at > 24 weeks) and disease activity at last visit suggest that a rapid response to TCZ seems to be related to long term inactivity of sJIA. 7/17 (41%) patients showing inactive disease at the last visit in 2014 had a response to TCZ within 5 weeks. Active disease in the current visit in 2014 was mostly seen in patients who did not show any response within an observation time of > 24 weeks (8/12, 67%).

### Different clinical course (Fig. 2)

Forty-six sJIA children were categorized into monocyclic (MC) 12/46 (26%), polycyclic (PC) 16/46 (35%) and polyarticular (PA) 18/46 (39%) disease courses. Comparison of these courses revealed significant differences in the outcome; PC showed the highest clinical response rate (81%, Odds ratio (OR) = 7.0, 95%CI: 1.8–27.2, *p* = 0.005) followed by the MC courses (59%, OR = 2.9, 95%CI: 1.1–8.6, *p* = 0.048) compared to the worst outcome for patients with PA courses (29%).

**Fig. 2:**
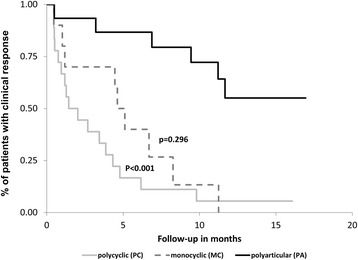
Clinical response with no clinical manifestation and normalized inflammation parameters at 12 weeks of treatment by disease courses

### Tolerance of TCZ and non-responders (Table 1)

Adverse events (AE) were analyzed for 46 patients contributing to 63 TCZ exposure years. Fourteen adverse events (22.2 AE/100 TCZ exposure years, 95%CI: 12.6–33.7) were reported in 11/46 (24%) patients including leukopenia, infections and elevated transaminases (at least two-fold increased), 2 serious adverse events (SAEs; 3.2 SAE/100 TCZ exposure years, 95%CI: 0.4–11.5) were one Hodgkin’s lymphoma [21] and one gut perforation [22]. No cases of MAS, amyloidosis, or death under TCZ were reported. In 2/46 MAS were diagnosed before TCZ in the medical history. In 5/46 (11%) adverse events were the reason for stopping TCZ administration: neutropenia *n* = 3, SAEs *n* = 2.

**Table 1:** Adverse events (AEs) and serious adverse events (SAEs) under tocilizumab (TCZ)

AEs and SAEs (*n* = 16) under TCZ in 11/46 patients	
Leukopenia	4
Recurrent infections	3
Elevated transaminases	2
Hypotonia	1
Hypertonia	1
Dyspnea	1
Abdominal pain	1
Lymphadenopathy	1
*Hodgkin’s-Lymphoma*	1
*Gut perforation*	1

Only 4/46 (9%) patients (three with PC, one with MC disease course) had to be defined as non-responders over the whole time. In 3/4 patients, symptoms got worse. They subsequently received canakinumab (CANA)/nonsteroidal anti-inflammatory drugs (NSAID), etanercept (ETA)/NSAID and anakinra (ANA)/MTX, respectively. Follow-up data from the fourth non-responder was not available.

### Concomitant therapy (Table 2)

Before starting TCZ therapy, 33/46 (**72%**) patients received disease-modifying antirheumatic drugs (DMARDs) (cyclosporine A (CSA), MTX, azathioprine (AZA), abatacept (ABA), colchicine (COL)), 24/46 (**52%**) biologicals (ANA, ETA, adalimumab (ADA), CANA, infliximab (IFX)), 23/46 (**50%**) corticosteroids (CO) and 21/46 (**46%**) NSAIDs. 6/46 (**13%**) children received no co-medication before TCZ.

**Table 2:** Co-medication before and during TCZ treatment (DMARD = CSA, MTX, AZA, ABA, COL, etc. = ANA, ETA, ADA, CANA, IFX, RTX)

Co-medication before TCZ		Co-medication during TCZ	
none	6/46 (13%)	TCZ mono	5/46 (11%)
CO mono	5/46 (11%)	TCZ + CO	4/46 (9%)
DMARD + etc	15/46 (33%)	TCZ + DMARD +etc	22/46 (48%)
DMARD + CO + etc	18/46 (39%)	TCZ + DMARD + CO + etc	14/46 (30%)
unknown	2/46 (4%)	unknown	1/46 (2%)
NSAID	20/46 (43%)	NSAID	17/46 (37%)

Co-medications at the initiation of TCZ were DMARDs (CSA, MTX, AZA) in 35/46 (**76%**) patients, NSAID in 16/46 (**35%**) and corticosteroids (CO) in 18/46 (**39%**) who received steroids in high- or low-dose and 3/18 (17%) who required additional steroid-pulses. 2/46 (**4%**) patients received ANA or rituximab (RTX) additionally to TCZ. In our patient population 21/46 (46%) had received TCZ as first biological treatment, 25/46 (54%) as second, third, fourth or fifth line therapy. Among those receiving TCZ as first line treatment, 14/21 (67%) showed ID or CRM after 12 months. The switch of biological agents to TCZ resulted in 14/25 (56%) additional cases in ID or CRM. In 9/46 patients a switch from TCZ to other biological agents was seen because of non-response (*n* = 4) or adverse effects (*n* = 5).

Over the whole period of TCZ therapy corticosteroids (CO) were discontinued in 50% (9/18) children. Under observation co-medication were used in 40/46 cases. Co-medications like DMARDs, corticosteroids (CO) and others were very variable and depended on the participating center. Monotherapy with TCZ was rare in responder (*n* = 2 and *n* = 1 according to response after 12 weeks and 12 months). After 12 weeks clinical responders (16/46) received TCZ + CO *n* = 3, TCZ + DMARD etc. *n* = 11, TCZ + DMARD etc. + CO *n* = 0 and only TCZ *n* = 2. After 12 months clinical responders (18/24) received TCZ + CO n = 1, TCZ + DMARD etc. *n* = 15, TCZ + DMARD etc. + CO n = 1 and only TCZ *n* = 1 (DMARD = CSA, MTX, AZA, ABA, COL, etc. = ANA, ETA, ADA, CANA, IFX, RTX).

## Discussion

sJIA is a severe multi-organ disease of unknown etiology. Classification and diagnostic criteria are currently under discussion because sJIA is not a classical autoimmune disease like most other subtypes of JIA but more related to AID. Only 74% of the patients included into the AID-registry fulfilled the ILAR criteria for sJIA while 26% were classified as sJIA based on other criteria [3, 17–20]. Usually sJIA is treated with various kinds of NSAIDs, and immunosuppressive drugs, but as an active disease frequently remains refractory. High-dose corticosteroids (CO) or combinations with MTX are often considered for therapy [23]. A primary aim of clinical research should be the avoidance or at least reduction of CO. Despite the availability of biological agents, one third of sJIA patients in Germany were still treated with systemic CO [14].

Long term follow-up in the AID-registry enables us to report first results on treatment, clinical response rate and tolerance of TCZ in a real-life setting. Another source of real-life data is the German Biker registry [24]. For this type of data, limitations were a small and heterogeneous population, missing data and an irregular recorded follow-up. In the past several clinical trials with TCZ have been reported for approval of TCZ in children with sJIA or withdrawal design to measure response rates during therapy with TCZ [12, 25]. Clinical trials are generally restricted to evaluating specific intervention with a focus on assessing the efficacy and safety of therapies in a highly selected and homogeneous population. Healthcare providers often underuse treatments due to a lack of data from patients who do not meet the clinical trial inclusion criteria. While collecting data in real-world settings can be challenging, understanding the effectiveness and safety of therapies in more severe and complicated disease courses can provide reassurance for clinicians [26].

Woerner et al. describe an achievement of inactive disease with maintenance in follow-up in 37/77 (48%) patients who had received biologicals without switching. This was observed in 33/61 patients on anti-IL-1 treatment and 2/2 on TCZ therapy. A switch to a second, third or fourth biological agent resulted in inactive disease in further 13/77 (17%) patients. Median follow-up duration on biological drug was 33.8 months for patients who did not experience a switch and 6.7 months for switchers [27]. In our patient population 21/46 (46%) had received TCZ as first biological treatment. Among those, 14/21 (67%) showed ID or CRM after 12 months. The switch of other biological agents to TCZ resulted in ID or CRM for an additional 14/25 (56%) cases. In 9/46 patients a switch from TCZ to other biological agents was seen because of non-response (*n* = 4) or adverse effects (*n* = 5). Kostik et al. showed that during TCZ treatment 40 out of 48 cases (83.3%) achieved remission within 5 months. Patients who achieved remission had milder disease courses [28].

Possible courses of sJIA are monocyclic (MC), polycyclic (PC) and polyarticular (PA) (arthritis > 4 joints) activity [29]. Patients with a PA course tend to lose systemic inflammatory activity, switching to a more autoimmune-like phenotype [30]. In our cohort, 12/46 (26%) children showed MC, 16/46 (35%) PC and 18/46 (39%) PA course. When applying a definition of first remission, Singh-Grewal et al. classified 42.2%, 20%, and 37.8% of the patients as having monophasic, polycyclic, and persistent disease [31]. These differences are explainable by a high degree of heterogeneity in the patient population for MC and PC phenotypes. In our cohort, outcome was worst in PA courses and best in PC courses. In case of predominant polyarticular arthritis (PA) and in case of lack of response to IL-1 or IL-6 inhibition, TNF-blockers were applied [5].

A large proportion of patients received MTX (89%) and CO (66%) before using TCZ [32]. In the second trial for approval of CANA all patients received CANA to taper steroids. In one third of the patients, CO could be discontinued, about half of the patients tolerated dose reduction [1, 10, 11]. With 9/18 (50%) cases discontinuing corticosteroids under TCZ, our rate was fairly high as compared to 19/155 (12%) from the Japan cohort [26]. Oligoarticular onset, absence of active arthritis, ESR < 26 mm/h and no requirement for CO therapy at 3 and 6 months appear to be predictive of a shorter time to remission [31]. IL-1 inhibition should be considered mainly in patients with high systemic disease activity and limited joint involvement, whereas TCZ seems to be appropriate in patients with extensive joint involvement [8, 33]. Vastert et al. started ANA in 20 patients with new-onset sJIA who were CO-naïve. At 3 months, 85% of patients achieved an adapted ACR Pedi 90 response or had inactive disease; 75% of patients achieved this response while receiving ANA monotherapy. In the majority of responding patients (73%), treatment could be stopped within 1 year, with remission being preserved during follow-up. However, in about one third of patients, concomitant therapy was required for maintenance of clinical response [34].

These findings have led to a shift in treating sJIA, with a recent tendency to use ANA, CANA and TCZ as first line therapy [35]. Woerner et al. postulate that introducing an IL-1 or an IL-6 inhibitor as a first biologic treatment dramatically increases the chance of sJIA patients to achieve clinical remission [7]. Our results suggest that CRM correlates with a fast response to TCZ within 5 weeks of treatment. Active disease was mostly seen in patients who did not show any response within 24 weeks. There was no benefit in the outcome if TCZ was applied as first line therapy. However, challenges in the management of sJIA remain, as 30% of patients continue to present with ongoing active disease [14].

As adverse effects, nasopharyngitis, respiratory tract infections, gastroenteritis, pharyngitis and elevation of transaminases have been described previously [26, 36, 37]. In our cohort, leukocytopenia was seen most often. Kessler et al. report three cases of children with sJIA who were treated with TCZ and developed cytopenia. Two of those children had a medical history of MAS suggesting that patients with a tendency towards MAS may have an increased risk of developing cytopenia when treated with TCZ [38]. We had two SAEs: The 15-year-old patient with multiple upper gastrointestinal perforations is published [22]. A 10-year-old girl with Becker myotonia and sJIA showed a rapid improvement of rheumatic symptoms by TCZ and steroids. After 11 months of treatment with TCZ a supraclavicular swelling was observed, histologically corresponding to lymphoma. Conceivably, at the time of sJIA diagnosis the underlying condition might already had been a Hodgkin lymphoma. As an alternative to independent occurrence of both diseases, a hypothetical causal link between TCZ and lymphoma as an AE was discussed. The Biker registry showed a risk ratio for AEs of 5.3/patient-year and for SAEs of 2.5/patient-year [24].

IL-6 induces elevation of acute phase reactants like CRP but also serum amyloid A, fibrinogen and ferritin [39, 40]. It has a key role in the pathogenesis, clinical manifestations and activity of sJIA. This is why under IL-6 inhibition CRP levels are often not measurable and cannot be used to monitor disease activity or to detect infections [41]. 27/46 (59%) of our patients show an active disease with CRP < 5 mg/l under therapy with TCZ. Shimizu et al. found that serum CRP never increased during TCZ therapy, even in MAS. In contrast, serum IL-6 concentrations increased during sJIA flare-up and with the complication of infection; serum IL-18 concentrations increased before clinical disease activity. The authors suggest monitoring serum concentrations of IL-18 and IL-6 for evaluation of disease activity in sJIA and to detect the complication of infection [42]. A correlation between increase of S100 proteins and sJIA has been suggested previously [43, 44].

## Conclusion

Reports on IL-6 blockade in sJIA are limited by relatively small patient numbers, diversity of study populations, highly heterogeneous treatment regimes, variable co-medications, and relatively short follow-up periods. Despite these shortcomings, TCZ appears to be effective in most patients. In our real-life clinical setting, we observed a clinical response rate of 35% after 12 weeks, a remission on medication in 54% and inactive disease in 21% after 12 months. If evaluated over the entire observation time, remission on medication was achieved in 30% and inactive disease in 39% of cases. It should be noted, however, that patients often require additional treatment. Fine-tuning of dosage and co-medication will need to be addressed by future long-term studies on larger patient cohorts.

## Abbreviations

AD: Active disease
ADA: Adalimumab
AE: Adverse events
AID: Autoinflammatory disease
ANA: Anakinra
AZA: Azathioprine
CANA: Canakinumab
CARRA: The Childhood Arthritis and Rheumatology Research Alliance
CO: Corticosteroids
CRM: Clinical remission under medication
CRP: C-reactive protein
CSA: Cyclosporine A
DMARDs: Disease-modifying antirheumatic drugs
DRFZ: German Rheumatism Research Centre
ESR: Erythrocyte sedimentation rate
ETA: Etanercept
GKJR: German society for paediatric rheumatology
i.v.: intravenous
ID: Inactive disease
IFX: Infliximab
IL-1, IL-6, IL-18: Interleukin-1, − 6, − 18
ILAR: International League of Associations for Rheumatology
JIA: Juvenile idiopathic arthritis
MAS: Macrophage activation syndrome
MC: Monocyclic
MTX: Methotrexate
NSAIDs: Nonsteroidal anti-inflammatory drugs
p.o.: per oral
PA: Polyarticular
PC: Polycyclic
PRINTO: Pediatric Rheumatology InterNational Trials Organisation
RTX: Rituximab
s.c.: subcutaneously
SAE: Serious adverse events
sJIA: Systemic juvenile idiopathic arthritis
TCZ: Tocilizumab
TNF: Tumor necrosis factor
